# The Revolution of Genetic Diagnosis: An Example from Rare Disorders

**DOI:** 10.3390/genes15101328

**Published:** 2024-10-15

**Authors:** Stefania Zampatti

**Affiliations:** Genomic Medicine Laboratory UILDM, IRCCS Fondazione Santa Lucia, 00179 Rome, Italy; s.zampatti@hsantalucia.it

Since the advent of DNA sequencing, genetic analyses have been increasingly incorporated into clinical practice to support the diagnosis of rare disorders [1]. Initially, the identification of specific loci and genes enabled geneticists to predict inheritance patterns and assess the recurrence risks of Mendelian genetic disorders. Today, advanced molecular and cytogenetic technologies—such as next-generation sequencing, genomic arrays, and epigenetic analyses—play a crucial role in supporting diagnostic protocols, offering timely diagnoses and early interventions [2,3,4]. Additionally, the discovery of new genetic causes of rare disorders is vital for improving diagnostic accuracy, refining genotype–phenotype correlations, and facilitating the development of new therapies [5,6]. Rare disorders characteristically affect less than one person in two thousand, and, to date, over 7000 rare disorders have been described, collectively impacting 300 million individuals worldwide [7]. Although the occurrence of each rare disorder is low, their overall prevalence is significant. In fact, it is estimated that 1 in 10 Americans is affected by a rare disease, a prevalence comparable to that of type 2 diabetes [8,9,10,11]. From an etiological perspective, approximately 70–80% of rare disorders have a genetic basis [2]. One of the primary challenges in healthcare for patients with rare disorders is providing a rapid and accurate diagnosis, which is crucial for the timely implementation of management protocols. Advances in genetic technologies for DNA analysis have introduced various multiomics approaches that enhance molecular diagnostics. While stratifying patients by phenotype can facilitate the targeted analysis of a select group of candidate genes, understanding the interactome and functional relationships between genes can lead to the discovery of new genes that are either primarily or secondarily implicated in rare diseases. In this Special Issue, about 200 authors contributed significant research, expanding the knowledge of rare disorders through a variety of approaches, ranging from the phenotype stratification, to the functional and interactome evaluation of genes.

In fact, the stratification by phenotype allowed Zhu and coworkers to perform a deep screening of the 22q11.2 genomic region in patients with microtia and congenital heart defects. The unexpected burden of genetic variations reported in this region supports the existence of a common genetic etiology (or locus) for this frequent association of phenotypes [12]. Another stratification by phenotype was performed by Saadi and coworkers. They reported nine consanguineous Pakistani families affected by rare spinocerebellar disorders, in which exome sequencing and homozygosity mapping allowed to define genetic signatures of rare spinocerebellar disorders [13]. Sometimes, the clinical evaluation of rare patients provides just a small amount of support in prioritizing genetic analysis, as is the case with very rare disorders with variable phenotypes, such as IMNEPD (infantile-onset multisystem neurologic, endocrine, and pancreatic disease). In this Special Issue, Sharkia and coworkers described clinical features of all IMNEPD patients described to date, with a peculiar evaluation of the *PTRH2* gene and its mutations, also providing a tentative genotype–phenotype relationship [14].

Knowledge of rare disorders is continuously promoted by both clinical case reports and fundamental research. In this Special Issue, Gallego-Zazo and her research team reported seven new patients with *SOX17*-related pulmonary arterial hypertension, also reviewing all *SOX17*-mutated patients reported in the scientific literature [15]. As is known, the SOX transcription factor family plays fundamental roles in controlling aspects of development. Through a gene-to-phenotype approach, Underwood and coworkers performed a systematic analysis of the evolutionary conservation of SOX genes and proteins, unraveling new insights in these pathways [16]. A similar but different approach was performed by Sarafidou and coworkers in their analysis of the FRA10AC1 interactome, confirming its role in spliceosomopathies and extending possible associated phenotypes [17].

Geneticists involved in the diagnosis of rare disorders should pay significant attention to different usual and unusual phenotypes. For example, there are many exceptions to Mendelian inheritance. In their review, Kalyta et al. reported clinical phenotypes in manifesting carriers of heterozygous variants in recessive genes [18]. Furthermore, the application of new genomic technologies in the diagnosis of rare disorders led to the production of a huge amount of genetic data that should be correctly interpreted and regularly re-evaluated over time. As elucidated by He and coworkers, many duplication variants historically reported in the *DMD* gene have been reclassified as benign [19].

In general, defining the complex scenarios underlying a rare disorder is mainly based on case reports. In this Special Issue, ten different case reports (two of which are in articles) were published, expanding phenotype knowledge of rare disorders [20,21,22,23,24,25,26], reporting differences when the multiple genetic determinants are recognized in the disease [27], or revealing new causative genes for known phenotypes [28,29].

After the well-known diagnostic odyssey, the management of rare disorders is an important problem as due to their rarity, the development of supportive therapies is difficult. In this scenario, the discovery of the CRISPR-Cas system has supported several new research strategies for the management of rare diseases. In this Special Issue, Sauvagère and Siatka provided a comprehensive review of CRISPR-Cas, from applications in fundamental research to diagnostics and therapeutic promises [30]. In order to support the management of patients affected by Fabry disease, Jaurretche and coworkers provided a review on biomarkers useful in monitoring renal damage [31].

In summary, in this Special Issue, different studies provided interesting evidence further expanding the knowledge of rare disorders. Seventeen articles and case reports were included to better elucidate the clinical and genotype presentation of rare disorders [12,13,14,15,16,17,19,20,21,22,23,24,25,26,27,28,29]. Three reviews focused on clinical presentation, therapeutic management, and molecular promises [18,30,31].

The study of rare diseases is highly complex, but a deep understanding of them enables us to provide vital support to millions of patients. Understanding rare disorders is much like assembling a complex puzzle. While we can often form a rough idea of the overall picture, the finer details remain unclear until every piece is in place. In the realm of rare diseases, each patient’s experience and willingness to participate in scientific research play a pivotal role. Every patient who consents to be part of a study or to be included in a case report adds a valuable piece to this intricate puzzle. Their contribution is not just a step toward their own diagnosis or treatment but also a crucial advancement in our collective understanding of the disorder. By filling in these gaps, they help refine diagnostic approaches, guide therapeutic development, and ultimately improve the medical care of others suffering from the same condition. In this way, each patient’s story becomes an essential part of the broader scientific effort to unravel the mysteries of rare diseases and provide hope for future breakthroughs.
